# Reduced argininosuccinate synthetase expression in refractory sarcomas: Impacts on therapeutic potential and drug resistance

**DOI:** 10.18632/oncotarget.12225

**Published:** 2016-09-23

**Authors:** Youngji Kim, Eisuke Kobayashi, Daisuke Kubota, Yoshiyuki Suehara, Kenta Mukaihara, Keisuke Akaike, Ayumu Ito, Kazuo Kaneko, Hirokazu Chuman, Akira Kawai, Shigehisa Kitano

**Affiliations:** ^1^ Division of Musculoskeletal Oncology, National Cancer Center Hospital, Tokyo, Japan; ^2^ Department of Experimental Therapeutics, National Cancer Center Hospital, Tokyo, Japan; ^3^ Department of Orthopaedic Surgery, Juntendo University School of Medicine, Tokyo, Japan; ^4^ Department of Hematopoietic Stem Cell Transplantation, National Cancer Center Hospital, Tokyo, Japan; ^5^ Exploratory Oncology Research and Clinical Trial Center, National Cancer Center, Tokyo, Japan

**Keywords:** argininosuccinate synthetase, bone and soft-tissue sarcoma, drug-resistance, metabolic error, P-glycoprotein

## Abstract

**Background:**

Treating drug-resistant sarcomas remains a major challenge. The present study aimed to identify a novel therapy for drug-resistant sarcomas based on metabolic errors involving argininosuccinate synthetase1 (ASS1).

**Results:**

ASS1 expression was reduced in Dox-resistant sarcoma cells. Immunohistochemistry and real-time PCR showed an inverse correlation between ASS1 and P-gp expressions. The inhibition of cellular proliferation with G1-arrest was shown to lead to autophagy with arginine deprivation. In addition, the combination of an autophagy inhibitor plus arginine deprivation was more effective than arginine deprivation alone. In cells with suppressed ASS1 expression, P-gp expression was upregulated as compared to that in negative controls.

**Discussion:**

These results indicate that the reduced ASS1 expression in Dox-resistant sarcomas may contribute to drug resistance in association with the expression of P-gp. ASS1 deficiency is a potential target for novel drug therapies. The combination of arginine-deprivation therapy and an autophagy inhibitor may have anti-tumor effects in refractory sarcomas.

**Methods:**

We assessed the expressions of ASS1 and P-glycoprotein (P-gp) in clinical specimens and cell lines of osteosarcoma (KHOS), doxorubicin (Dox)-resistant osteosarcoma (KHOS_R2_), epithelioid sarcomas (ES-X and VAESBJ) and alveolar soft part sarcoma (ASPS-KY). Each cell line was cultured in arginine-containing and arginine-free media. Cell growth was assessed using an XTT assay and flow cytometry. We analyzed the induction of autophagy in arginine-free medium. Moreover, we assessed the expression of P-gp after suppressing ASS1 in Dox-sensitive cells (MCF-7 and KHOS) and after transfecting ASS1 into Dox-resistant cells (ES-X, VAESBJ, ASPS-KY and KHOS_R2_).

## INTRODUCTION

The prognosis of patients with sarcomas has improved since the introduction of chemotherapy in the 1980s [1]. Doxorubicin (Dox) is one of the key agents currently used to control advanced bone and soft-tissue sarcomas [2, 3]. However, some bone and soft-tissue sarcomas initially have or develop resistance to currently available chemotherapeutic agents and this remains a major clinical challenge. In particular, alveolar soft part sarcoma (ASPS) and epithelioid sarcoma (ES) are characterized by being soft-tissue sarcomas unresponsive to the antitumor drug regimens presently used in clinical settings. ASPS, which was initially described by Christopherson in 1952, is a very rare soft-tissue sarcoma of unknown histogenesis that most commonly occurs in adolescents and young adults [4]. In previous reports, ASPS was noted to be a soft-tissue sarcoma in which cytotoxic chemotherapy had little effect [5]. Although complete excision of the primary tumor is the only curative treatment option [6, 7], most patients already have metastatic dissemination (especially to the lungs and brain) at the time of diagnosis. In such inoperable cases, the outcomes are very poor [8]. ES, which was first proposed by Enzinger in 1970 [9], tends to develop in the hands and fingers of young adults [10]. It predominantly affects the subcutaneous tissues, fasciae or the tendon sheaths of the hands and forearms [9, 11]. The prognosis of ES patients is reported to be relatively poor because ES is characteristically resistant to chemotherapy [7, 8, 11], metastasizes to the regional lymph nodes and has a local recurrence rate of 65% or higher [10]. In contrast, osteosarcoma (OS) is the most frequent primary malignant bone tumor in children and young adults. The introduction of preoperative combined chemotherapy in the last three decades has significantly improved the survival rate of OS patients to approximately 70% [12]. However, the outcomes of OS patients who show a poor response to chemotherapy remain unfavorable due to their high risk of developing distant metastasis. Thus, the identification of a potential target that would allow the development of novel therapeutic options remains an urgent issue in efforts to improve the outcomes of patients with drug-resistant sarcomas.

Metabolic dysregulation is often critical to the growth and progression of cancer [13]. Recently, argininosuccinate synthetase 1 (ASS1) has attracted interest as a potential therapeutic target based on the advantages of using arginine auxotrophy. ASS1 is one of the metabolic enzymes required for the biosynthesis of arginine, a semi-essential amino acid, through the urea cycle. Reduced ASS1 expression is frequently detected in various human cancers including sarcomas [14–23].

In the present study, we investigated whether reduced ASS1 expression is a potential therapeutic target in refractory sarcomas and the relevance of the association between ASS1 and P-glycoprotein (P-gp) expressions.

## RESULTS

### Cytotoxic effects of Dox on sarcoma cells

To evaluate the cytotoxicity of Dox, XTT assays were performed for MCF-7 and sarcoma cells. The 50% inhibitory concentration (IC_50_) values for colony formation are shown in Table 1. The IC_50_ values for MCF-7 and KHOS cells were 0.09μM and 0.22μM, respectively. The IC_50_ values for the KHOS_R2_, VAESBJ, ES-X, and ASPS-KY cells were approximately 0.98μM, 13.03μM, 5.52μM and 10.91μM, respectively. These results revealed MCF-7 and KHOS to be Dox-sensitive cells. In contrast, VAESBJ, ES-X, ASPS-KY, and KHOS_R2_ were found to be Dox-resistant cells.

**Table 1:** IC_50_ of Doxorubicin for each cell line

	IC_50_ (uMol)
MCF-7	0.09
VAESBJ	13.03
ES-X	5.52
ASPS-KY	10.91
KHOS	0.22
KHOS_R2_	0.98

IC_50_:Drug concentration for 50% reduction in colony formation.

### Expressions of P-gp and ASS1 in Dox-resistant sarcoma cells

**Figure 1:**
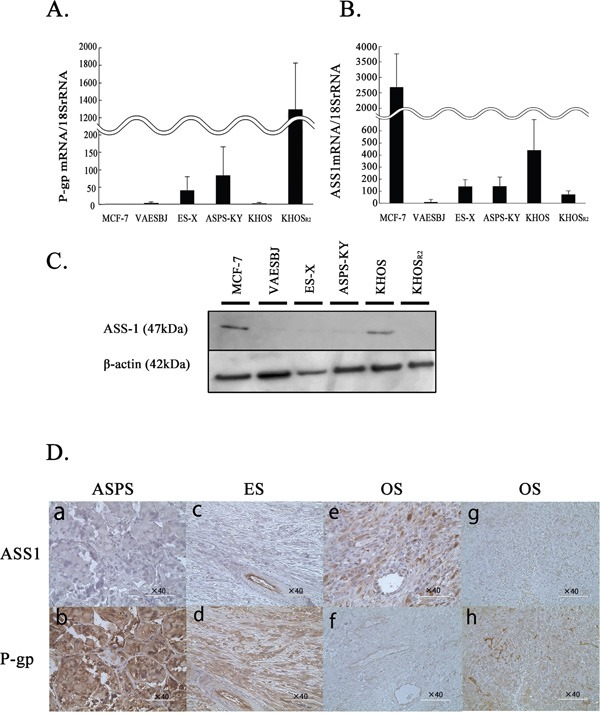
The levels of P-gp and ASS1 expression in MCF-7 cells and five sarcoma cells **A.** The P-gp mRNA expression was determined by real-time PCR. **B.** ASS1 mRNA expression was determined by real-time PCR. **C.** ASS1 protein expression in cells was evaluated by Western blotting. **D.** ASS1 (a, c, e, g) and P-gp (b, d, f, h) immunohistochemical staining was performed on paraffin-embedded sarcoma specimens. Tissue samples were obtained from ASPS patient: case1 (a, b), ES patient: case5 (c, d), OS patient who with a good response to chemotherapy: case7 (e, f) and OS patient with a poor response to chemotherapy:case11 (g, h).

P-gp plays an important role in drug transport and is related to chemotherapy resistance in various malignancies. Thus, we confirmed P-gp expression levels using the real time polymerase chain reaction (PCR) method. In comparison to Dox-sensitive cells (MCF-7 and KHOS), P-gp mRNA expression was upregulated in all Dox-resistant cells (VAESBJ, ES-X, ASPS-KY and KHOS_R2_ cells**)** (Figure 1A). It is noteworthy that KHOS_R2_ cells showed the highest expression of P-gp.

The expression of ASS1 was also confirmed in each cell line by real-time reverse transcription-PCR (Figure 1B). The ASS1 mRNA expression levels were reduced in Dox-resistant as compared to Dox-sensitive cells. Similarly, ASS1 protein expression was shown to be upregulated by Western blotting (Figure 1C).

To assess the clinical relevance of the association between ASS1 and P-gp expressions, we confirmed these expressions immunohistochemically in an independent cohort from patients with ASPS, ES and OS (Figure 1D, Table 2). Of the 15 OS patients, 5 patients showed good and 10 poor responses to chemotherapy. While ASS1 expression was negative in all ASPS and ES samples (Figure 1Da and Figure 1Dc), P-gp expression was positive in all samples (Figure 1Db and Figure 1Dd). As to the OS patients, ASS1 expression was positive in 80% (4/5) of good responders (Figure 1De) and in 10% (1/10) of poor responders. P-gp expression was positive in 40% (2/5) of good responders and in 90% (9/10) of poor responders (Figure 1Dh). In summary, Dox-resistant sarcomas showed higher P-gp and lower ASS1 expression than Dox-sensitive cells.

**Table 2:** Relationships of clinical and histological characteristics of sarcoma patients with ASS1and P-gp expressions

	Case	Sex	Age	ASS1	P-gp	Chemotherapy response
ASPS	1	F	30	−	+	NA
	2	M	32	−	+	NA
ES	3	F	75	−	+	NA
	4	M	47	−	+	NA
	5	F	51	−	+	NA
OS	6	M	11	+	−	good
	7	M	14	+	−	good
	8	F	59	−	+	poor
	9	M	12	−	−	poor
	10	M	7	−	−	good
	11	M	13	−	+	poor
	12	F	8	−	+	poor
	13	F	10	+	+	good
	14	F	13	+	+	good
	15	M	18	−	+	poor
	16	M	16	−	+	poor
	17	F	12	−	+	poor
	18	F	14	+	+	poor
	19	F	14	−	+	poor
	20	M	10	−	+	poor

ASPS: Alveolar soft part sarcoma, ES: Epithelioid sarcoma, OS: Osteosarcoma

M: Male, F: Female.

*NA: Not available*.

### Cell growth inhibition caused by arginine deprivation in Dox-resistant sarcomas

**Figure 2:**
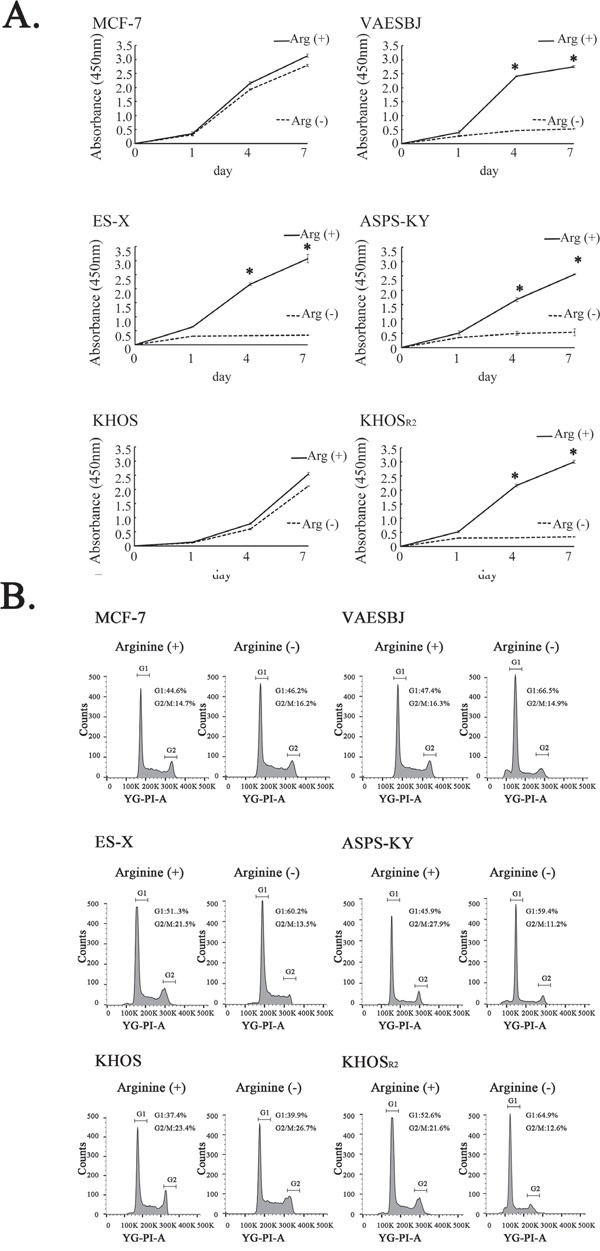
Effects of arginine deprivation on cellular proliferation and the cell cycle in MCF-7 cells and five sarcoma cell lines **A.** The effects of arginine deprivation on the proliferation of MCF-7 cells and the five sarcoma cell lines were measured employing XTT assays. * P < 0.05 in comparison to the control group (arginine-containing medium). **B.** The cell cycles were analyzed by the FACS analysis method under arginine-deprivation conditions in MCF-7 cells and the five sarcoma cells.

XTT assays were conducted to observe cell growth in arginine-containing and arginine-free media. No obvious changes were detected in response to arginine deprivation in either MCF-7 or KHOS cells. In contrast, the Dox-resistant cells in which the expression of ASS1 was relatively reduced showed cell growth inhibition in response to arginine deprivation (Figure 2A).

To elucidate the mechanisms underlying the cell growth inhibition associated with arginine deprivation, a fluorescence-activated cell sorting (FACS) analysis was carried out. The FACS analysis revealed the MCF-7 and KHOS cells that cultured in arginine-containing medium to not differ significantly from those cultured in arginine-free medium. An increase in the proportion of cells in the G1 phase and a decrease in the proportion of those in the G2-M phase were observed in the Dox-resistant cells when cultured in arginine-free medium. These results indicate arginine deprivation to induce G1 arrest in Dox-resistant sarcoma cells with low levels of ASS1 expression (Figure 2B).

### Arginine deprivation caused autophagy in Dox-resistant sarcoma cells

**Figure 3:**
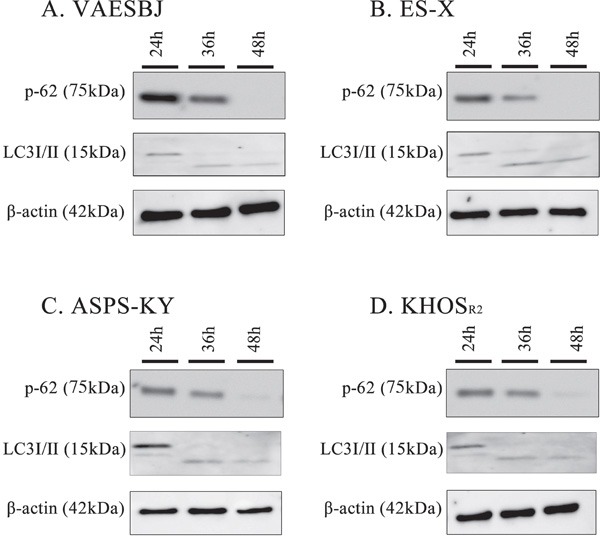
Arginine deprivation induces autophagy in Dox-resistant sarcoma cells Dox-resistant sarcoma cells **A.** VAESBJ, **B.** ES-X, **C.** ASPS-KY, **D.** KHOS_R2_ cells) were cultured in arginine-free medium and analyzed by Western blotting to identify autophagy markers (P62 degradation and the conversion of LC3-1 to LC3-11.

The levels of p62 and LC3-I/II expression were investigated by Western blotting to examine the relationship between autophagy and arginine deprivation. In Dox-resistant sarcoma cells, the autophagic changes were detectable based on the time-dependent degradation of p62 and the conversion of cytosolic LC3-I/II to the lipidated form (Figure 3). Given these observations, we can reasonably speculate that arginine deprivation caused the autophagic change in Dox-resistant sarcoma cells.

### Chloroquine (CQ) inhibits autophagy and augments the cell death induced by arginine deprivation

**Figure 4:**
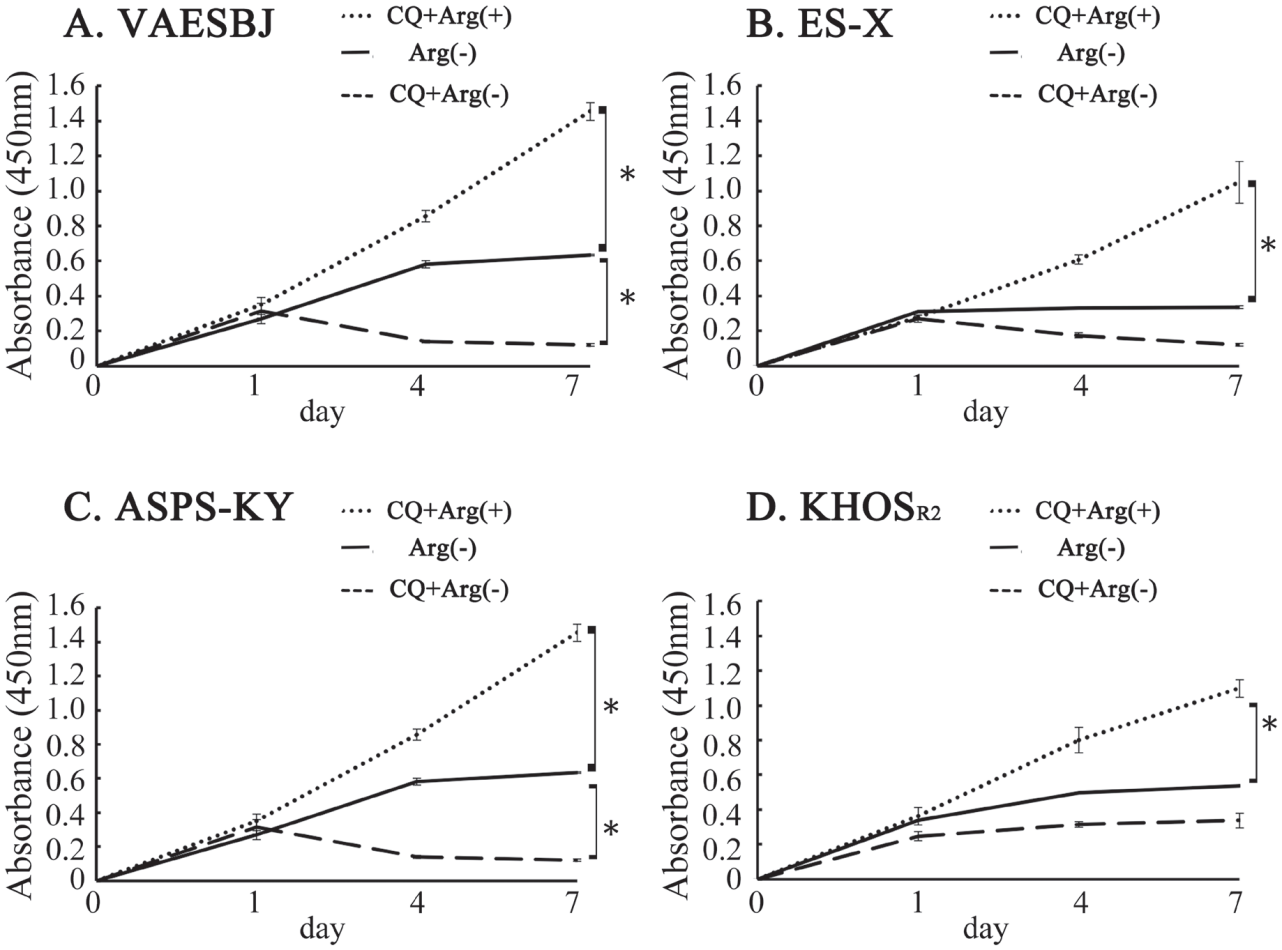
Cellular proliferation was inhibited in Dox-resistant sarcoma cells by arginine deprivation and accelerated by CQ Effects on Dox-resistant sarcoma cell proliferation in arginine-containing medium with CQ added, arginine-free medium and arginine-free medium with CQ added were measured employing an XTT assay **A.** VAESBJ, **B.** ES-X, **C.** ASPS-KY, **D.** KHOS_R2_ cells.

XTT assays were conducted to determine whether the growth of Dox-resistant cells was promoted by arginine-free medium, arginine-containing medium with CQ (10μM) or arginine-free medium with CQ. The cells grown in arginine-containing and arginine-free media with CQ were compared, and cell growth inhibition was found to be far more marked in those grown in arginine-free medium with CQ (Figure 4).

### Upregulation of P-gp expression in response to suppression of the ASS1 gene

**Figure 5:**
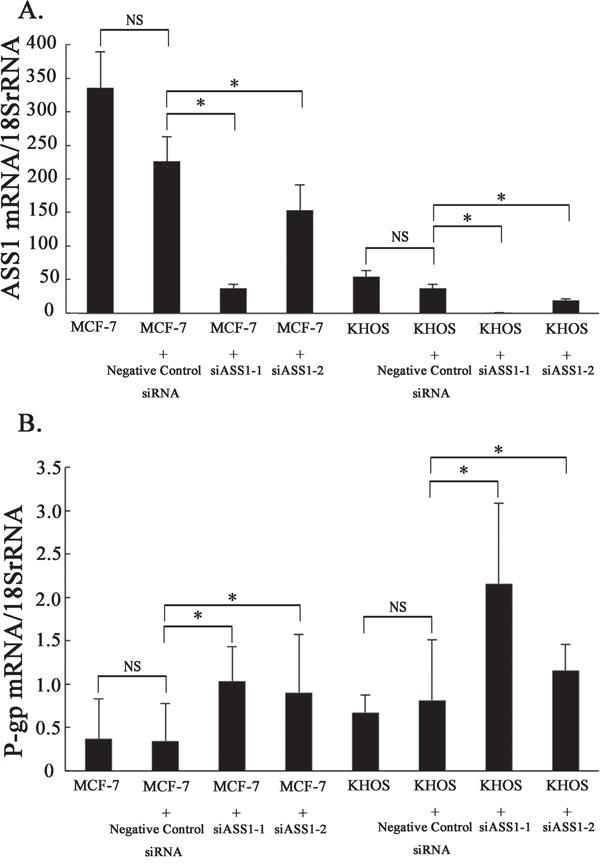
P-gp mRNA expression after the knockdown of ASS1 in Dox-sensitive cells **A.** The levels of ASS1 mRNA expression in ASS1-expressing cells (MCF-7, MCF-7 with negative control siRNA, MCF-7 with siASS1, KHOS, KHOS with negative control siRNA and KHOS with siASS1) were determined by real-time PCR. **B.** P-gp mRNA expression in ASS1-expressing cells was determined by real-time PCR.

In MCF-7 and KHOS cells in which the ASS1 gene had been silenced, real time PCR showed P-gp mRNA expression to be significantly upregulated as compared to that in controls (Figure 5A, 5B). In Dox-resistant sarcoma cells in which P-gp expression had been suppressed, however, ASS1 mRNA expression did not change (Supplementary Figure S1A-S1D). Dox-resistant cells which had been transfected with ASS1 showed no change in P-gp mRNA expression. (Supplementary Figure S1E-S1H).

## DISCUSSION

Dox is a key drug for treating OS and soft-tissue sarcomas and belongs to the family of anthracycline antitumor agents [24–26]. However, in the clinical setting, we often encounter patients whose sarcomas have become Dox-resistant due to the development of multidrug resistance (MDR). Therefore, the development of MDR remains a major challenge in managing patients with OS and soft-tissue sarcomas including ASPS and ES [27].

To the best of our knowledge, no prior studies examined whether or not ES and ASPS are resistant to chemotherapy *in vitro*. We confirmed these cells to be Dox-resistant *in vitro,* based on the IC_50_ of Dox being significantly higher in these cells than in Dox-sensitive cells (MCF-7 and KHOS) (Table 1).

Drug resistance in cancer is frequently associated with P-gp overexpression. P-gp is the gene product of the MDR protein 1 genes (*MDR1, ABCB1*) and a drug efflux pump that prevents the intracellular accumulation of anthracycline drugs [28, 29]. P-gp overexpression is associated with poor outcomes in patients with OS and soft-tissue sarcomas [30, 31]. Thus, P-gp expression was examined in each of these cells. P-gp expression was elevated in Dox-resistant cells as compared to Dox-sensitive cells (Figure 1A).

ASS1 is one of the metabolic enzymes required for the biosynthesis of arginine through the urea cycle. Arginine is widely recognized as a semi-essential amino acid that can be taken up extracellularly when cells cannot produce arginine autonomously [32, 33]. ASS1-deficient cancers rely on extracellular arginine, due to arginine auxotrophy [34]. Arginine deiminase (ADI) was developed from a mycoplasma-derived enzyme as a novel anticancer enzyme to induce arginine deprivation [35]. Pegylated arginine deiminase (ADI-PEG20) is pegylated to reduce its immunogenicity and cause tumor cell death by selectively inducing arginine deprivation. Based on the advantages of using arginine auxotrophy in tumors, arginine deprivation has been investigated as a novel therapeutic strategy aimed at disrupting cancer metabolism [14–16, 20, 21, 36–40]. With regard to sarcomas, in OS, reduced ASS1 expression is not only a novel predictive biomarker for the development of metastasis, but also a potential target for pharmacologic intervention [22]. ASS1 deficiency due to promoter methylation is associated with poor outcomes of patients with myxofibrosarcoma and ADI-PEG20 has been suggested as a possible therapeutic target [23]. However, the expression and roles of ASS1 in Dox-resistant sarcomas have not yet been investigated. In the present study, we confirmed ASS1 expression to be reduced in all Dox-resistant sarcoma cells (Figure 1B, 1C). Furthermore, we also demonstrated the association between ASS1 and P-gp expressions in clinical samples of ASPS, ES and OS (Figure 1D). In all ASPS, ES and chemo-resistant OS samples, ASS1 expression was essentially negative, but P-gp expression was positive except 1 chemo-resistant OS sample (Table 2). Interestingly, our results suggest an inverse correlation between the expressions of ASS1 and P-gp in both cell lines and clinical samples. Because ASS1 expression was reduced in Dox-resistant sarcoma cells, we conducted cell proliferation assays for each of the cells examined under arginine-deprivation conditions. Dox-resistant sarcoma cells in arginine-free medium showed inhibited cell growth with G1 arrest, while the growth was unaffected in arginine-containing medium (Figure 2, Table 3). These findings raise the possibility that arginine deprivation may be a new therapeutic target in patients with Dox-resistant sarcomas.

**Table 3:** Cell cycles of each cell line in arginine-containing and arginine-free medium

	Arginine (+)		Arginine (−)	
	G1 (%)	G2/M (%)	G1 (%)	G2/M (%)
MCF-7	44.6	14.7	46.2	16.2
VAESBJ	47.4	16.3	66.5	14.9
ES-X	51.3	21.5	60.2	13.5
ASPS-KY	45.9	27.9	59.4	11.2
KHOS	47.4	23.4	49.9	26.7
KHOS_R2_	52.6	21.6	64.9	12.6

Arginine (+):arginine-containing medium, Arginine (−): arginine-free medium.

Generalized amino-acid deprivation has been suggested to induce autophagy [41]. Similarly, arginine deprivation promoted autophagy as a means of survival in ASS1-deficient cancers [42, 43]. Autophagy, which involves the self-digestion of cells that are subjected to stress and starvation, is an important cellular catabolic process for survival [44, 45]. In this study, arginine deprivation appeared to induce autophagy in Dox-resistant cells (Figure 3). Therefore, we hypothesized that autophagy inhibition in response to arginine deprivation might serve as the basis for developing novel therapies for Dox resistant sarcomas. CQ, which is used for the treatment of malaria, is known to be an autophagy inhibitor that blocks the fusion of lysosomes [15, 46]. We conducted cell proliferation assays in Dox-resistant cells by combining CQ with arginine deprivation. This combination was found to inhibit cell growth more effectively than arginine deprivation alone (Figure 4). These findings raise the possibility of using this combination as a novel therapy for Dox-resistant sarcomas. To the best of our knowledge, this is the first report to describe autophagy induction, in sarcoma cells, in response to arginine deprivation.

Although the reason for reduced ASS1 expression in certain types of cancer that generally have a poor prognosis remains to be elucidated, reduced ASS1 expression has been noted to be associated with resistance to chemotherapy. In pancreatic cancer, reduced ASS1 expression predicts unfavorable tumor behaviors such as lymph node metastasis, local invasion and resistance to gemcitabine-based chemotherapy [17]. In ovarian cancer, the loss of ASS1 expression specifically confers resistance to platinum-based chemotherapy [47]. We hypothesized that ASS1 expression in Dox-resistant sarcoma might be associated with resistance to chemotherapy because the level of P-gp expression showed an inverse correlation with that of ASS1. In this study, P-gp expression was significantly enhanced by suppressing that of ASS1 in Dox-sensitive cells (Figure 5A, 5B). However, ASS1 expression was not enhanced by suppressing that of P-gp in Dox-resistant cells (Supplementary Figure S1A-S1D). In cells transfected with ASS1, P-gp expression was not reduced (Supplementary Figure S1E-S1H). The inverse correlation between ASS1 and P-gp expressions in both cell lines (Figure 1A–1C, Figure 5A–5B) and clinical samples (Figure 1D) raise the possibility that ASS1 expression contributes to chemotherapy resistance in association with the expression of P-gp in Dox-resistant cells, although the correlation between ASS1 and P-gp expressions may not be direct.

The present study has limitations. First, we cannot conclude that our *in vitro* results are applicable to *in vivo* settings (animal experiments). Furthermore, the drug resistance associated with P-gp expression may be only one of several factors contributing to drug resistance. Thus, we need to design future studies to identify other factors possibly contributing to the development of drug resistance.

In conclusion, we demonstrated ASS1 expression to be reduced in Dox-resistant sarcoma cells. We hypothesize that this reduction contributes to the development of drug resistance, which is known to be related to P-gp expression. Our results also suggest that the reduced ASS1 expression might serve as a target for novel pharmacological interventions, even in patients with Dox-resistant sarcomas. As the induction of autophagy in response to arginine deprivation may have a pro-survival role in patients with ASS1-deficient sarcomas, the combination of arginine deprivation therapy with autophagy modulators might potentiate anti-tumor effects in patients with drug-resistant sarcomas. We anticipate that validation of these results will lead to clinical applications in the treatment of refractory bone and soft-tissue sarcomas.

## MATERIALS AND METHODS

### Cell culture

The two epithelioid sarcoma cell lines (ES-X and VAESBJ), were kindly provided by Dr. Tsukahara (Sapporo Medical University Hospital, Hokkaido, Japan). Alveolar soft part sarcoma cells (ASPS-KY) were kindly provided by Dr. Miyagi (Kanagawa Cancer Center Research Institute, Kanagawa, Japan). The OS cells (KHOS and KHOS_R2_) were kindly provided by Dr. Duan (Massachusetts General Hospital, MA, US). The breast cancer cells (MCF-7) were obtained from Exploratory Oncology Research, National Cancer Center Hospital (Tokyo, Japan).

The VAESBJ and ASPS-KY cells were cultured in Dulbecco's modified Eagle's medium (Thermo Fisher Scientific, Massachusetts, US). The ES-X cells were cultured in Iscove's Modified Dulbecco's Medium (Thermo Fisher Scientific). The MCF-7, KHOS and KHOS_R2_ cells were cultured in RPMI 1640 (Thermo Fisher Scientific). All of the cells were incubated at 37°C in a humidified 5% CO_2_ atmosphere supplemented with 10% fetal bovine serum (Thermo Fisher Scientific). In addition, the VAESBJ cells were cultured with Non-Essential Amino Acids (Thermo Fisher Scientific). Arginine-free medium was used as a substitute for arginine deprivation therapy in the present study. Arginine-free medium was prepared by the Cell Science & Technology Institute (Miyagi, Japan) and was supplemented with 10% dialyzed fetal bovine serum (Thermo Fisher Scientific).

Dox was purchased from Cell Signaling Technology Japan (Tokyo, Japan). Chloroquine (CQ) was purchased from Sigma-Aldrich (MO, USA). To analyze the cytotoxicity of Dox and CQ, MCF-7, VAESBJ, ES-X and KHOS cells were seeded into 96-well plates at a density of 3,000 cells per well and then incubated for 24 h. The ASPS-KY and KHOS_R2_ cells were seeded into 96-well plates at a density of 4,000 cells per well. Incubation was continued for 72 h after Dox treatment, at which point 100 μl of the medium were replaced with fresh medium.

### Patients and tumor samples

With institutional review board (IRB) approval (IRB No.2004-050), all samples were obtained by biopsy from the primary tumor sites of 2, 3 and 15 patients with ASPS, ES and OS, respectively, at the National Cancer Center Hospital (Tokyo, Japan) between 2011 and 2016. With regard to OS, the response to chemotherapy was classified as good if the extent of tumor necrosis was at least 90%.

### Immunohistochemical analysis

Formalin-fixed paraffin-embedded sections of ASPS, ES and OS were deparaffinized and dehydrated. The sections were then subjected to antigen retrieval in an autoclave. The sections were incubated with mouse monoclonal antibody against ASS1 (Abnova, Neihu District, Taipei) at a 1:200 dilution and P-Glycoprotein / MDR-1 (Thermo Fisher Scientific, Hudson, NH, USA) at a 1:1000 dilution. Staining was visualized using the DAB detection system (Dako, Carpinteria, CA, USA).

### Cellular proliferation assays

Cellular proliferation was examined employing an XTT-based colorimetric assay according to the manufacturer's protocol. Briefly, each of the cells was cultured in 96-well plates. After incubation periods of 1, 4 and 7 days, a Cell Proliferation Kit XTT based Colorimetric Assay (Biological Industries, Kibbutz Beit-Haemek, Israel) was added to each well and incubation was continued for 6 hours at 37°C. Optical density was measured at a wavelength of 450 nm using a 96-multiwell microplate reader (TECAN, Mannedorf, Switzerland).

### Real time PCR

Total RNA was extracted using a Pure Link RNA Mini kit (Thermo Fisher Scientific). For the synthesis of cDNA, 1 μg of total RNA was reverse transcribed using a Super Script VILO cDNA Synthesis Kit (Thermo Fisher Scientific). The primers and TaqMan probes for 18S rRNA, ASS1 and P-gp were obtained from Applied Biosystems (TaqMan Gene Expression Assays). The amplification data, measured as increases in reporter fluorescence, were collected using a 7500 Fast Real-Time PCR system (Applied Biosystems). The mRNA expression level relative to the internal control (18S rRNA gene) was calculated using the comparative threshold cycle method.

### Western blotting

The cells were cultured in a 75 cm^2^ flask until they reached approximately 80% confluence. Total cellular protein was extracted by scraping the cells into M-PER™ Mammalian Protein Extraction Reagent (Thermo Fisher Scientific). The cellular proteins were centrifuged at 15000 rpm for 30 minutes at 4°C, and then placed on ice for 30 minutes. The protein concentration was measured using a BSA protein assay kit (Bio-Rad, CA, USA). Ten-microgram portions of the protein samples were separated by SDS-PAGE and transferred onto the membranes. After blocking at room temperature for 1 h with skim milk, incubation with primary antibodies against β-actin (1:1000 dilution Abcam, Cambridge, MA, USA), SQSTM1 / p62 (1:500 dilution, Abcam), ASS1 (1:500 dilution, Abnova, Neihu District, Taipei), and LC3-I/II (1:1000 dilution, Abcam) and the relevant secondary antibodies (Anti-Mouse IgG antibody and Anti-Rabbit IgG antibody) (GE Healthcare Japan, Tokyo, Japan), was continued at room temperature for 1 h. Blots were detected using enhanced chemiluminescence Western blotting detection reagents (Bio-Rad).

### Fluorescence activated cell sorting assay

Cells were harvested using trypsinization and then centrifuged at 1,000 rpm for 5 min. A Cycle TEST PLUS DNA Reagent kit (Becton Dickinson, NJ, USA) was used to stain the cells. The DNA content was analyzed using an LSR Fortessa X-20 system (Becton Dickinson).

### Gene silencing assay

ASS1- and P-gp-specific siRNAs were purchased from Sigma-Aldrich, and control stealth siRNA was obtained from Life Technologies. The target sequences were 5′-rGrCrAUUrAUUUrGrArCrCrArGrArGUUTT-3′, 5′-rCrArGrArGUUrGrArArGUrGrArCrArGrArATT-3′, and 5′-rGrCrAUUrAUUUrGrArCrCrArGrArGUUTT −3′. In total, 5 × 10^3^ cells were seeded into each well of a 96-well plate (Coaster, Cambridge, MA, USA). The following day, the cell monolayer was washed with pre-warmed sterile phosphate-buffered saline. The cells were transfected with the appropriate siRNA using Lipofectamine transfection reagents (Thermo Fisher Scientific) in accordance with the manufacturer's protocol. Twenty-four hours later, the culture medium of the transfected cells was replenished. Total protein and RNA were extracted at 72 hours after transfection.

### Establishment of ASS1-overexpressing cell lines

The plasmid pCMV6-ASS1 carrying the human full-length ASS1 cDNA sequence (GenBank, NM_000050; Origene technologies, Rockville, MD, USA) and an empty vector (pCMV6) were transfected into VAESBJ, ES- X, ASPS-KY and KHOSR2 cells using the transfection reagent lipofectamine 3000 (Thermo Fisher Scientific).

## SUPPLEMENTARY FIGURE


