# Supplementary material for: Reduced argininosuccinate synthetase expression in refractory sarcomas: Impacts on therapeutic potential and drug resistance
Source: Oncotarget. 2016 Sep 23;7(43):70832–44. doi: 10.18632/oncotarget.12225 (PMC5342592; doi:10.18632/oncotarget.12225)
Supplement: Supplementary file 1 [file oncotarget-07-70832-s001.pdf]

# Reduced argininosuccinate synthetase expression in refractory sarcomas: Impacts on therapeutic potential and drug resistance

## SUPPLEMENTARY FIGURE

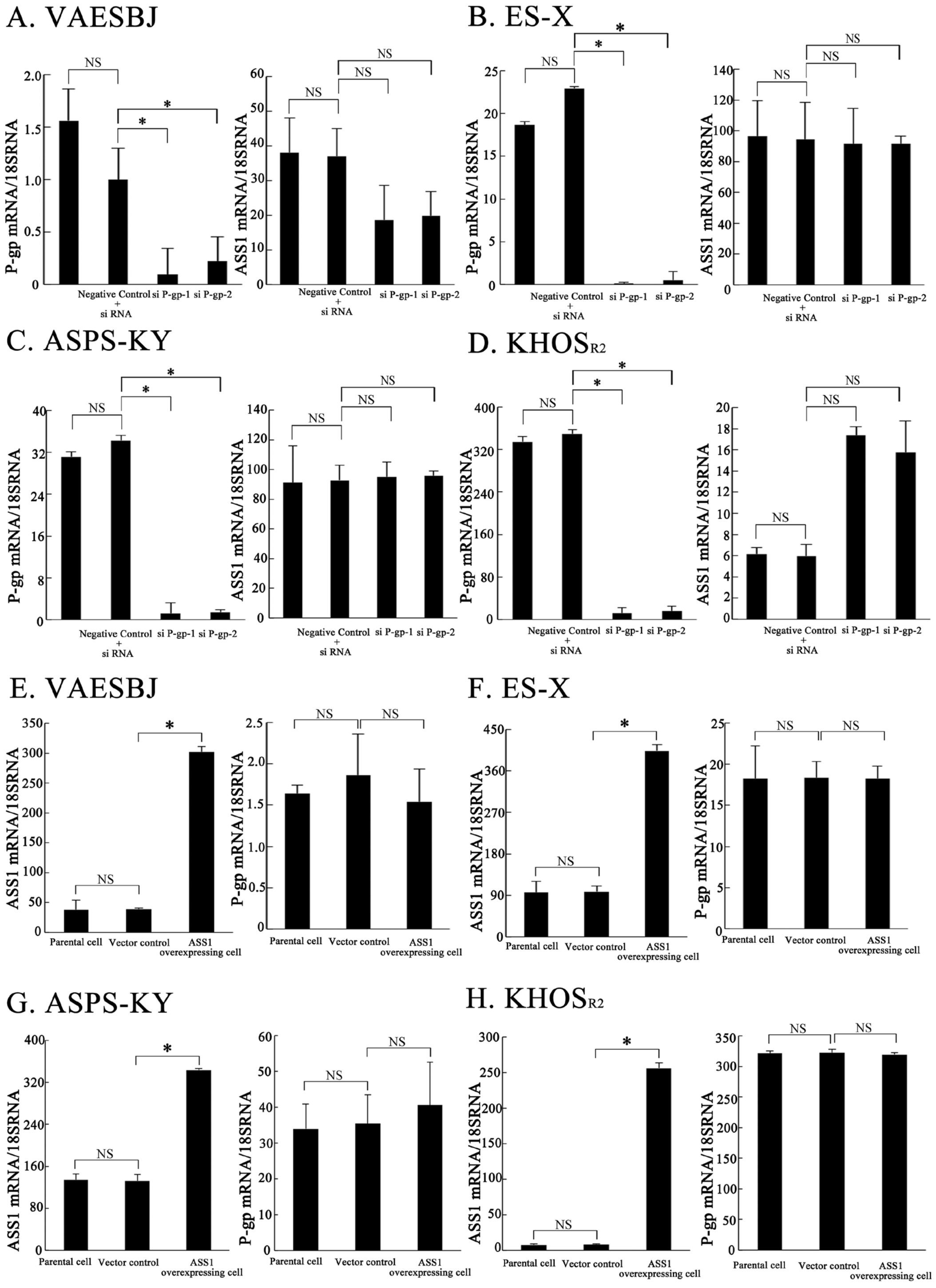


**Supplementary Figure S1: A-D.** ASS1 mRNA expression after the knockdown of P-gp in Dox-resistant cells and **E-H.** P-gp mRNA expression in Dox-resistant cells with induction of ASS1.
